# Targeting Tumor Microenvironment: Effects of Chinese Herbal Formulae on Macrophage-Mediated Lung Cancer in Mice

**DOI:** 10.1155/2017/7187168

**Published:** 2017-05-28

**Authors:** Fei Xu, Wenqiang Cui, Zhengxiao Zhao, Weiyi Gong, Ying Wei, Jiaqi Liu, Mihui Li, Qiuping Li, Chen Yan, Jian Qiu, Baojun Liu, Jingcheng Dong

**Affiliations:** ^1^Department of Integrative Medicine, Huashan Hospital, Fudan University, Shanghai, China; ^2^Institutes of Integrative Medicine, Fudan University, Shanghai, China; ^3^Department of Integrative Medicine and Neurobiology, State Key Laboratory of Medical Neurobiology, Institute of Acupuncture Research, School of Basic Medical Science, Fudan University, Shanghai, China

## Abstract

Our previous studies have shown that Qing-Re-Huo-Xue (QRHX) formulae had significant anti-inflammatory effects in chronic airway diseases such as asthma and chronic obstructive lung disease. Here, we examined the effects of QRHX on lung cancer cell invasion and the potential associated mechanism(s), mainly polarization of macrophages in the tumor microenvironment. In vivo, QRHX both inhibited tumor growth and decreased the number of tumor-associated macrophages (TAMs) in mice with lung cancer. Further study indicated that QRHX inhibited cancer-related inflammation in tumor by decreasing infiltration of TAMs and IL-6 and TNF-*α* production and meanwhile decreased arginase 1 (Arg-1) expression and increased inducible NO synthase (iNOS) expression. QRHX could markedly inhibit CD31 and VEGF protein expression. Additionally, CXCL12/CXCR4 expression and JAK2/STAT3 phosphorylation were reduced in QRHX treatment group. Thus, we draw that QRHX played a more important role in inhibiting tumor growth by regulating TAMs in mice, which was found to be associated with the inhibition of inflammation and the CXCL12/CXCR4/JAK2/STAT3 signaling pathway.

## 1. Introduction

Lung cancer is the most common cause of cancer-related deaths in men and women globally and with the highest rate of morbidity and mortality [1]. In 2014, about 1.5 million new patients were diagnosed worldwide and approximately 1.6 million die of this disease every year [2]. Non-small-cell lung cancer (NSCLC), accounting for 80% of lung cancer, has a dismal five-year survival at 15% and even worse, patients with advanced NSCLC, if left untreated, have a median survival of 4-5 months after diagnosis [2, 3]. During the past decades, new treatments, such as minimally invasive, surgery, targeted therapy, adjuvant chemotherapy, and individualized therapy, have been applied but have a less important effect in improving the overall 5-year survival, especially in the advanced stage [4]. Nonetheless, all above therapies have focused on tumor cells.

However, uncontrolled growth in tumors, invasion, and metastasis cannot be elucidated solely by aberrations in cancer cells themselves. Over the past few decades, a major paradigm shift happens to cancer therapy and a great deal of effort has been put forth to develop therapies that target the tumor microenvironment. Accumulating evidences suggest that the alterations that occur in the stroma around a tumor prove useful in antiangiogenesis, antitumor metastasis, and prognosis [5]. Nontumoral cells, including stromal cells (fibroblasts, endothelium cells, etc.) and leukocytes, are a prominent component of solid tumors [6]. Tumor-associated macrophages (TAMs), an important component, acquire a distinct, tissue-specific phenotype in different microenvironments and have both anti- and protumor effect due to two distinctly different polarization, respectively, referred to as “classical” (or M1) and “alternative” (or M2) activation [7]. In tumor microenvironment, TAMs are primarily polarized toward a M2-like phenotype, which have the ability to promote the growth and vascularization of tumors. Collective evidences demonstrate that the dual roles of TAMs have been demonstrated both in vitro and in vivo in different tumor models [8]. Moreover, clinical studies make strong cases that TAMs, characterized by M2 phenotypes, are poor predictors of prognosis and progression in numerous malignancies [9].

TAMs also profoundly influence the effects of conventional treatment modalities (chemotherapy and radiotherapy), targeted drugs, antiangiogenic agents, and immunotherapy, including checkpoint blockade [10]. Therefore, TAMs are essential for effective therapy and M2-like TAMs are considered to be potential target for adjuvant anticancer therapies. In addition, extensive studies have been carried out to declare that approaches targeting M2-like TAMs have gained encouraging results. Thus there is a growing appreciation that skewing TAM polarization away from the M2- to M1-like phenotype is of great importance.

Accumulated data indicates that traditional Chinese medicine (TCM) plays a pivotal role in regulating tumor microenvironment, including remodeling immunosuppressive microenvironment, hypoxia microenvironment, angiogenesis/lymphangiogenesis, and extracellular matrix [11]. Qing-Re-Huo-Xue (QRHX) formulae consist of a 1 : 1 mixture (w/w) of Radix Paeoniae Rubra and* Scutellaria baicalensis*. The above formula is frequently used in treatment of chronic inflammatory diseases in the respiratory system and immunocompromised diseases in TCM. Increasing evidence revealed that QRHX and its components, extracts, and derivative have the ability of anticancer and anti-inflammation [12–14]. Nevertheless, there are no reports which concerned the alleviated effects of QRHX on macrophage-mediated lung cancer. In the present study, we aimed to investigate the relevance between macrophage polarization and the antitumor effect of QRHX in mice.

## 2. Methods

### 2.1. Animal

Male C57BL/6J mice (5 weeks old) were purchased from Shanghai SLAC Co. (Shanghai, China) and housed in separate stainless steel cages (six mice per cage) at constant temperature (23°C) with a 12 h light/dark cycle had free access to water and food. All procedures of this study were approved by the Fudan University Animal Care and Use Committee (number 2015000518547).

### 2.2. Reagents

The following antibodies were used: VEGF, abcam46160 and abcam1613; CXCR4, abcam124824; CXCL12, abcam25117; p-JAK, CST3717; CD31, abcam28364; inducible NO synthase (iNOS), abcam15323; arginase 1 (Arg-1), CST385; CD11b, abcam1211; CD206, abcam64693 and abcam8918; Alexa Fluor 488, ALEXA21202 and 594 and ALEXA21207.

### 2.3. QRHX Preparation Chemical Constituents Identification

QRHX, a two-herb Chinese medicinal formula, is comprised of Radix Paeoniae Rubra and* Scutellaria baicalensis*. QRHX granules (batch number: 1211301) were prepared and supplied by Jiangyin Tianjiang Pharmaceutical Co. Ltd. Briefly, their component herbs were admixed in the prescribed proportion, which were soaked in distilled water (1 : 10 w/v) for 2 h and extracted at 100°C for twice (1 h each time). Then the decoction was filtered and concentrated to the extract with a relative density 1.13 at 60°C. After spray drying, the drying powder was blended thoroughly and made into 18–40 mesh particles. The granules were stored at 4°C and dissolved in distilled water of double volume before use. To ensure standardization and maintain interbatch reliability of QRHX, chemical ingredients of QRHX were separated and identified by high-performance liquid chromatography quadrupole time-off light mass spectrometry ultraviolet (HPLUNG CANCER-Q/TOF MS-UV). Briefly, 8 chemical components were, respectively, identified as major material basis in QRHX (Supplementary Figure 1 and Table 1 in Supplementary Material available online at https://doi.org/10.1155/2017/7187168).

### 2.4. Cell Culture

Lewis lung cancer (LLC) cells lines were cultured in DMEM supplemented with 10% Hyclone Fetal Bovine Serum (FBS; ThermoFisher Scientific, Fremont, CA, USA) in an atmosphere of 95% oxygen and 5% CO_2_ at 37°C. The cells were grown in 75 cm^2^ culture flasks and harvested in a solution of trypsin-EDTA at the logarithmic growth phase.

### 2.5. Subcutaneous Models and Drugs Administration

LLC cells were harvested by a brief treatment with trypsin/EDTA and then resuspended in DMEM with 10% FBS. Then cells were washed with cold PBS by centrifugation and resuspended in PBS to the concentration of 1 × 10^7^/mL and kept on ice before used. The male mice were randomly divided into two groups (*n* = 12, each group), including NS and QRHX groups. Tumor cells (2 × 10^6^ cells in 0.2 mL PBS) were injected subcutaneously into the right of the back. After 10 days, tumor size was measured twice weekly by a digital caliper and was estimated as (*D*^2^ × *d*)/2, where *D* is the large diameter and *d* is the small diameter of the tumor. Twenty-four hours after establishing model, mice were administered by intragastric (i.g.) in 0.2 mL volume for 24 consecutive days to different groups with QRHX and normal saline (NS) respectively and then sacrificed at day 24 after injection.

### 2.6. Enzyme-Linked Immunosorbent Assay (ELISA)

Concentrations of serum IL-6 and TNF-a were measured using an ELISA. The blood sample was stored at room temperature for 2 h, centrifuged (5000 rpm) for 30 min, and then cryopreserved at −80°C. The concentrations of IL-6 and TNF-a were measured using a sandwich ELISA kit (Multisciences, China).

### 2.7. Flow Cytometric Analysis

Tumor tissue was smeared, pushed through 200 mesh screen twice, and then resuspended by PBS. The suspension was treated with erythrocytolysin and then wash by PBS twice and finally suspended by PBS. Cells were then fixed and stained with PerCP-Cyanine 5.5-labeled anti-mouse CDD45, FITC labeled anti-mouse CD11b, Antigen PE labeled anti-mouse F4/80, and Alexa Fluor 647 labeled anti-mouse CD206 antibodies according to the manufacturer's instructions followed by detection by a FACSCalibur instrument (BD Bioscience).

### 2.8. Western Blot Analysis

Total protein was extracted from the cells using a RIPA kit (Beyotime, China). Cell debris was removed by microcentrifugation, and supernatants were quickly frozen. The protein concentration was determined by BSA method. And then the protein was electrophoresed on a polyacrylamide gel and transferred to a PVDF membrane. Next, the membranes was incubated 1 h–1.5 h with 5% milk at room temperature and then incubated overnight at 4°C with a 1 : 1000 or 1 : 500 dilution of corresponding primary antibodies. Blots were again washed three times with Tris-buffered saline/Tween 20 (TBST) and then incubated with a 1 : 5000 dilution of HRP-conjugated secondary antibody for 1 h at room temperature. Blots were again washed three times with TBST and then developed by enhanced chemiluminescence. Band intensities were quantified using UN-SCAN-IT gel analysis software (version 6).

### 2.9. QT-PCR

Total RNA was extracted with Trizol reagent (Ambion, Thermo), and reverse transcriptionwas performed to obtain the cDNA using the PrimeScript RT Reagent Kit (Takara, Japan), according to the manufacturer's protocol. The primers used were synthesized by (Sangon Biotech, China). These queues were as follows: iNOS: 5′-GTTCTCAGCCCAACA ATACAAGA-3′ (forward) and 5′-GTGGACGGGTCGATGTCAC-3′ (reverse); IL-6: 5′-GATACCACTCCCAAC AGAC-3′ (forward) and 5′-CTTTTCTCATTTCCACGAT-3′ (reverse); CCL22: 5′-AGGAAGGC TTGGCTTTTAGG-3′ (forward) and 5′-TGGTACCTTGCAGGCTCTCT-3′ (reverse); TNF-*α*: 5′-ACGGCATGGATCTCAAAGAC-3′ (forward) and 5′-GTGGGTGAGGAGCACGTAGT-3′ (reverse); GAPDH: 5′-AAATGGTGAAGGTCGGTGTG-3′ (forward) and 5′-AGGTCAATG AAGGGGTCGTT-3′ (reverse). Quantitative real-time PCR (QT-PCR) was performed using SYBR green reaction mixture in the ViiA™ 7 Real Time PCR System (Bio-Rad, MiniOpticon). The relative expression levels were calculated using 2−ΔΔCt methods.

### 2.10. Immunofluorescence (IF)

Cryostat sections were fixed and permeated. CD11b and CD206 antibodies for mice tumor tissue were used, followed by Alexa Fluor 488 (ALEXA) or 594 (ALEXA).

### 2.11. Immunohistochemical Analysis

IHC stain was performed using a two-step EnVision/HRP technique according to the manufacture's instruction. For three slides, cytoplasm stained with brown was scored as positive. The expression of CD206, CD31, and VEGF was quantitatively evaluated using Olympus Cx31 microscope with Image-Pro Plus medical image analysis system. The digital images were captured using a digital camera (Canon A640). The positive area and OD of CD206 and VEGF positive cells were evaluated by Image-J software and determined by measuring three randomly selected microscopic fields for each slide. The IHC index was defined as average integral optical density (AIOD) (AIOD = positive area × OD/total area).

CD31, a marker factor in vascular endothelial cell, was positively correlated with microvascular density (MVD). Then we counted the MVD by detecting the expression of CD31 antibody in tumor tissues by immunohistochemistry method. Firstly, at low power field (×40), three most intense tissue sections were selected each slice and then at high power field (×100), MVD counts of these areas were evaluated. Finally, the mean microvessel counts of the three most vascular areas were regarded as MVD.

### 2.12. Statistical Analyses

Data were from three independent experiments and expressed as mean ± SEM. Statistical analyses were performed by the one-way analysis of variance (ANOVA) for differences among different groups. About comparison of two groups, Student's *t*-test was used. All analyses were undertaken using GraphPad Prism6. *P* < 0.05 was considered statistically significant.

## 3. Results

### 3.1. QRHX Inhibits Tumor Growth in a Subcutaneous Mouse Model

**Figure 1:**
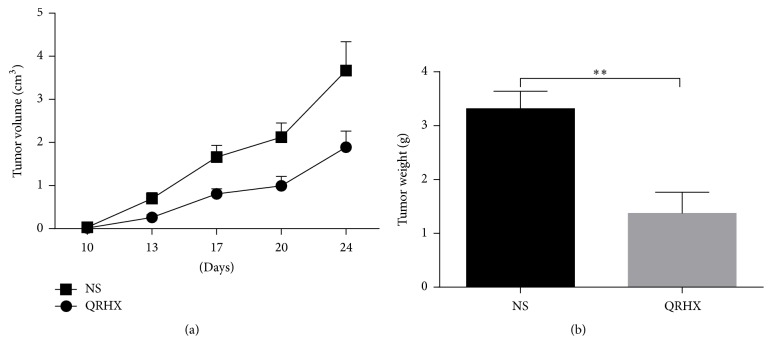
*QRHX inhibits growth of tumor in a subcutaneous mouse model*. C57/BL6 mice were subcutaneously injected with LLC. QRHX reduced tumor volume (a) and weight (b). The data represent means ± SEM (*n* ≥ 10). Compared with NS group, ^*∗∗*^*P* < 0.01.

In order to explore the role of QRHX in tumor growth in vivo, subcutaneous mouse model was established by subcutaneous injection of LLC in left extremity auxiliary. Mice were sacrificed at the end of treatment. As shown in Figure 1, the tumor volumes were performed on days 10, 13, 17, 20, and 24. In NS group, the tumor volume gradually increased in a time-dependent manner (Figure 1(a)). However, treatment with QRHX significantly suppressed the tumor volume (Figure 1(a)). The final tumor weight on day 24 after the start of treatment showed a significant decrease in the QRHX group compared with NS control (Figure 1(b), *P* < 0.01). These data suggested that QRHX could dramatically inhibit tumor growth in vivo.

### 3.2. QRHX Suppresses Cancer-Related Inflammation in Lung Cancer

**Figure 2:**
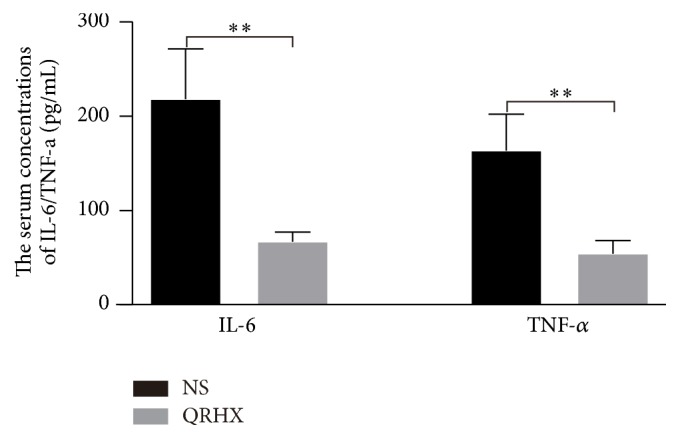
*QRHX suppresses cancer-related inflammation in lung cancer*. A subcutaneous mouse model was established and treated with QRHX or NS as described above. Blood was collected from each mouse, and the serum concentrations of the proinflammatory cytokines IL-6 and TNF-a were detected by ELISA. Student's *t*-test was used to determine the statistical significance, ^*∗∗*^*P* < 0.01.

Numerous studies have indicated that cancer-related inflammation promotes the development of tumor [15, 16]. In addition, some proinflammatory cytokines, such as IL-8, IL-6, and TNF-a, have been shown to ultimately facilitate cell invasion and metastasis [15]. Therefore, we used an ELISA assay to examine serum levels of the two proinflammatory cytokines IL-6 and TNF-*α* in peripheral blood. Serum IL-6 and TNF-*α* levels were lower in mice treated with QRHX compared with those receiving NS treatment (Figure 2, both *P* < 0.01). These data demonstrated that QRHX decreased the production of proinflammatory cytokines.

### 3.3. QRHX Reduces the Accumulation of TAMs in Lung Cancer

**Figure 3:**
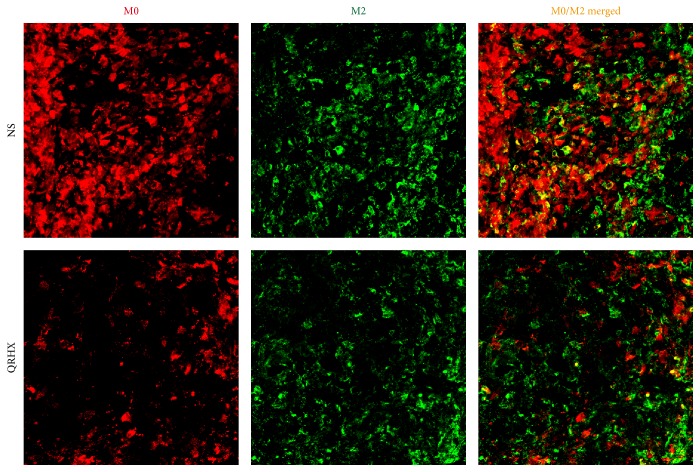
*Immunofluorescence staining for CD11b and CD206 of tumor tissues in subcutaneous tumor*. Mice with subcutaneous tumor were dealt with NS and QRHX. Images are at magnification of 200x. The expression of M0 and TAMs (M2-like macrophage) was, respectively, analyzed using an Alexa Fluor-549- and Alexa Fluor-488-conjugated secondary antibody.

**Figure 4:**
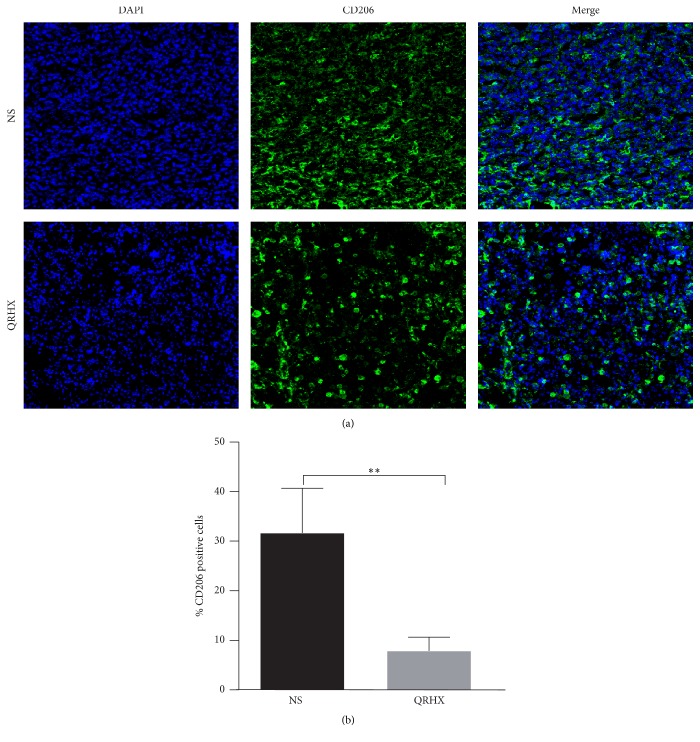
*Immunofluorescence staining for CD206 of tumor tissues in subcutaneous tumor*. Mice with subcutaneous tumor were dealt with the group of NS and QRHX. Images are at magnification of 200x. The expression of TAMs was, respectively, analyzed using an Alexa Fluor-488-conjugated secondary antibody. The nuclei were stained with DAPI. Compared with NS group, ^*∗∗*^*P* < 0.01.

**Figure 5:**
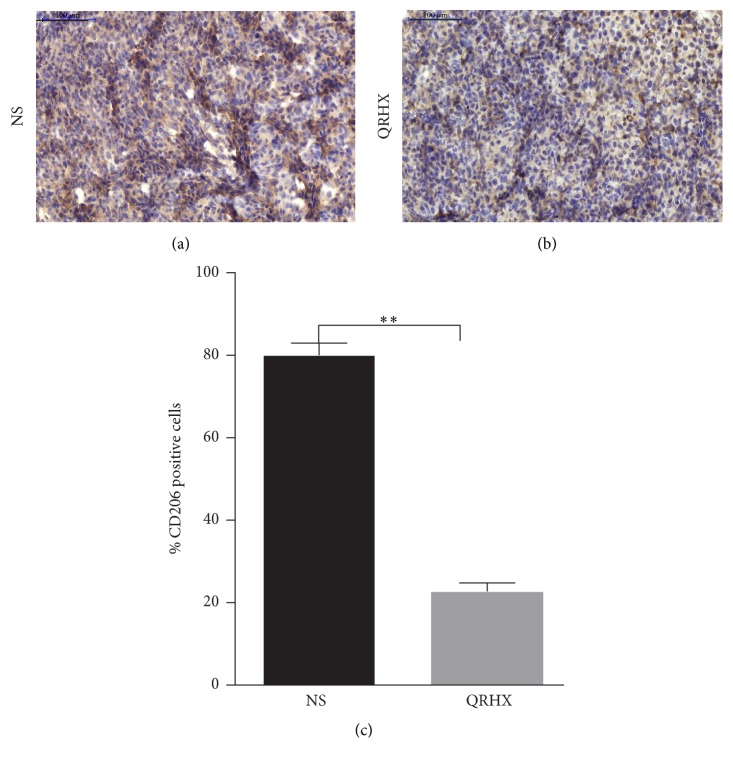
*Comparison of CD206+ macrophages infiltration between NS and QRHX groups*. Immunohistochemical staining of CD206+ macrophages in lung cancer tissues. Images are at magnification of 200x. Data represent mean ± SEM; *n* = 4. Compared with NS group, ^*∗∗*^*P* < 0.01.

**Figure 6:**
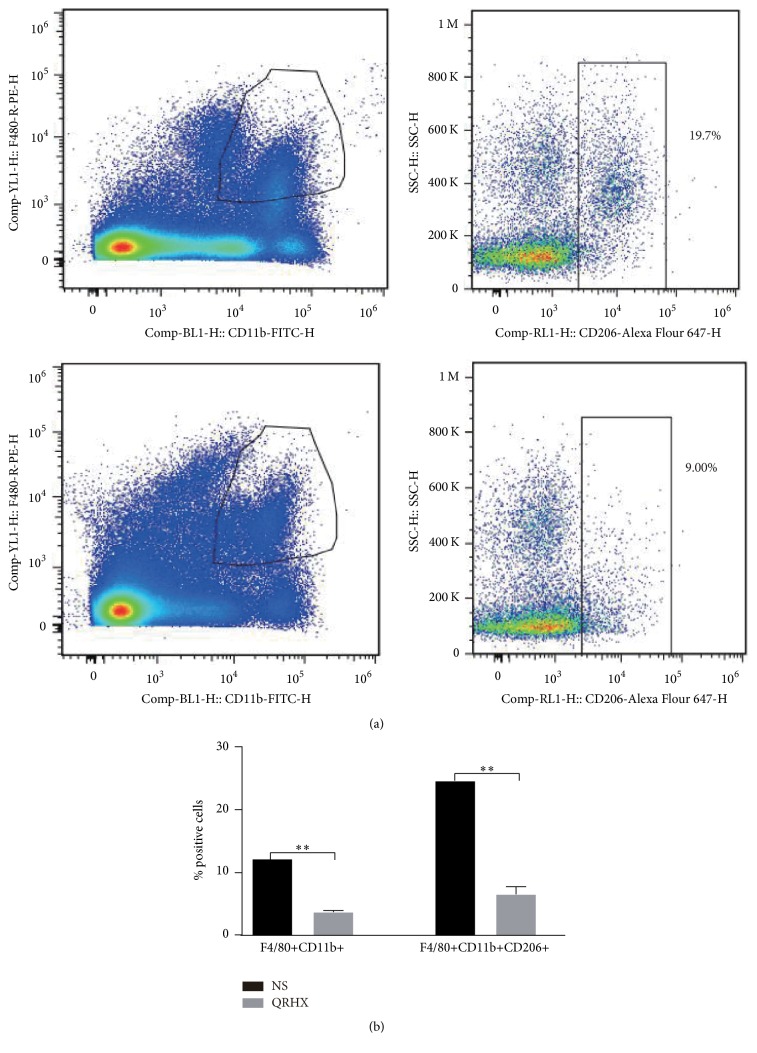
*QRHX decreases the number of TAMs in subcutaneous tumor*. Mice with subcutaneous tumors were dealt with NS and QRHX. Macrophages and TAMs (M2-like subtype) in tumor tissue were measured by flow cytometry. Data represent mean ± SEM; *n* = 4. Compared with NS group, ^*∗∗*^*P* < 0.01.

In order to ascertain the effects of the QRHX on TAMs, M2-like macrophage phenotype, we used flow cytometry, IF, and IHC assay to examine CD206 expression. As shown in Figures 3–6, our results revealed that CD206 expression was dramatically expressed in tumor tissue. Compared to NS group, oral administration with QRHX led to a prominent inhibition in TAMs (Figures 3–6, *P* < 0.01). These data demonstrated that QRHX had a more significant role in impeding accumulating and reducing the number of TAMs in lung cancer.

### 3.4. QRHX Alters Hallmarkers of M1 and M2 Macrophage in Lung Cancer

**Figure 7:**
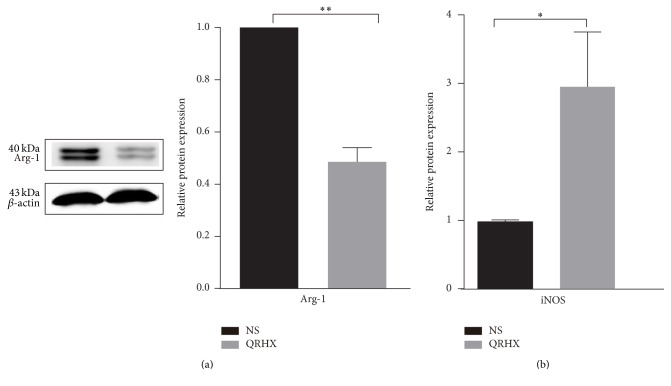
*The expression of M2-related marker (Arg-1) and M1-related marker (iNOS) was detected by western blot and QT-PCR, respectively*. Mice with subcutaneous tumor were dealt with NS and QRHX. Data were presented as means ± SEM (*n* = 3). Compared with NS group, ^*∗*^*P* < 0.05; ^*∗∗*^*P* < 0.01.

To further confirm the role of QRHX in TAMs, we analyzed the level of Arg-1 protein, M2 marker, and the mRNA expression of M1 marker iNOS. After QRHX treatment, Arg-1 level decreased (Figure 7(a), *P* < 0.01) and iNOS level increased (Figure 7(b), *P* < 0.05). Accordingly, these results strongly supported the inhibitory effect of QRHX on suppressing TAMs infiltration and regulating M2-like macrophage polarization.

### 3.5. QRHX Inhibits Angiogenesis in Lung Cancer

**Figure 8:**
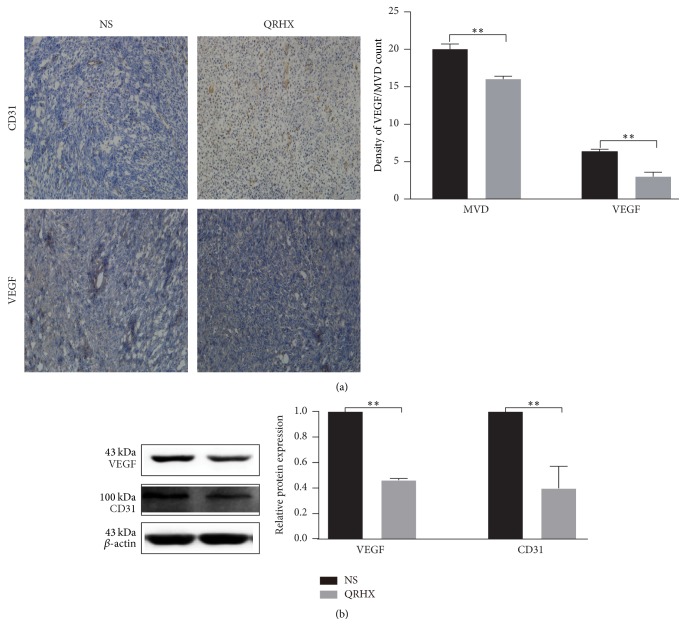
*QRHX inhibits angiogenesis in lung cancer*. Mice with subcutaneous tumors were dealt with NS and QRHX. (a) The nucleus was dyed as blue; CD31 was dyed as brown. MVD were performed at high power field (×200). (b) The VEGF and CD31 protein expression in tumor tissues was detected by western blot. Data was expressed as means ± SEM values (*n* = 4). Compared with NS group, ^*∗∗*^*P* < 0.01.

CD31, endothelial cell surface antigen, is a vascular endothelial marker for MVD and TAMs have a positive effect on blood vessels by inducing the production of various proangiogenic genes [17]. Therefore, CD31 was evaluated in implanted tumors using IHC and WB. MVD stained by anti-CD31 was measured by counting tissue sections of central areas of the tumor. As shown in Figure 8, QRHX treatment induced a remarkable reduction in MVD and CD31 protein (*P* < 0.01). Furthermore, IHC and WB demonstrated reduced vascular endothelial growth factor (VEGF) expression in the QRHX treated groups, relative to NS controls (Figure 8, *P* < 0.01). Taken together, these data clearly showed that QRHX reduced angiogenesis in lung cancer.

### 3.6. QRHX Blocks CXCL 12/CXCR4/JNK2/STAT3 Signaling Pathways

**Figure 9:**
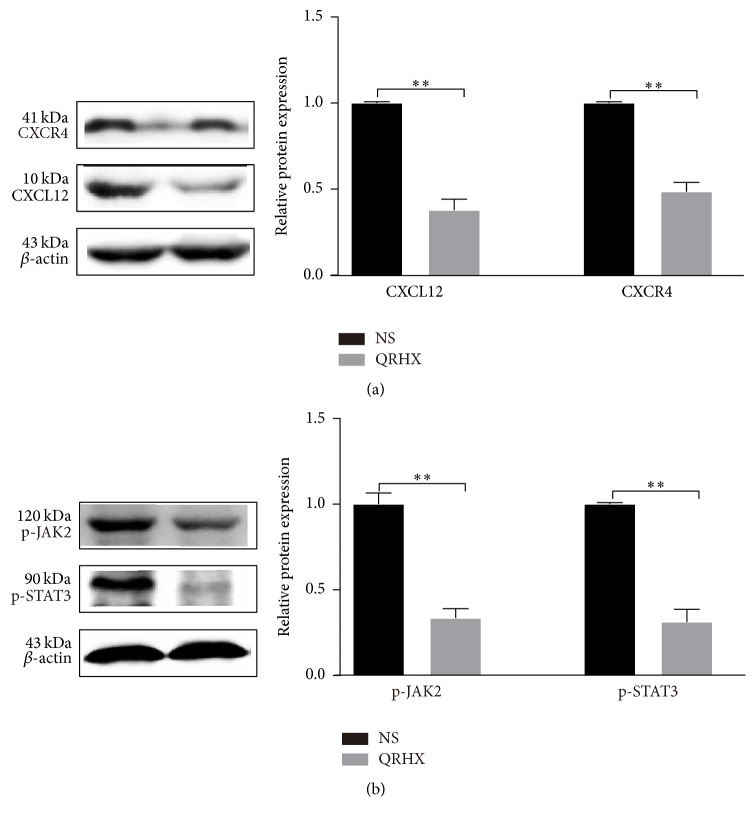
*QRXH regulates TAMs by inhibiting the CXCL12/CXCR4/JAK2/STAT3 signaling pathways*. Mice with subcutaneous tumor were dealt with NS and QRHX. Representative images of western blot and densitometry analysis showing the expressions of CXCL12, CXCR4, p-JAK2, and p-STAT3 in tumor. Compared with NS group, ^*∗∗*^*P* < 0.01.

To investigate the molecular mechanism underlying the formulae decreasing M2 macrophages, we tested the effects of inhibitors of signaling molecules. CXCL12/CXCR4 and JNK2/STAT3 are known to be important molecules involved in M2 polarization [18, 19]. Compared to NS group, oral administration of QRHX dramatically attenuated the increased expressions of CXCL12 and CXCR4 in tumor tissue (Figure 9(a), *P* < 0.01). As shown in Figure 9(b), JAK2 and STAT3 activation was significantly suppressed by QRHX treatment (both, *P* < 0.01) compared to NS group. Taking together, the data suggested that QRHX inhibited tumor cell-TAMs interactions possibly through blocking CXCL12/CXCR4/JAK2/STAT3 signaling pathways and then regulated macrophages polarization.

## 4. Discussion

In the present study, our data demonstrated that QRHX played a more crucial role in inhibiting tumor growth by modulating tumor microenvironment, especially TAMs. QRHX inhibited tumor cell-TAMs interactions via the suppression of cancer-related inflammation and probably blocking the response of macrophages to tumor signals, CXCL12/CXCR4/JAK2/STAT3 axis.

In the nineteenth century, the association between cancer and inflammation was firstly put forward [20]. Large number of studies provided powerful evidence that chronic inflammation can promote tumor development, progression, and metastasis, as well as chemoresistance [16, 21]. Recent studies have confirmed that paeoniflorin, baicalein, and wogonin, important ingredients of QRHX, had the potential to inhibit many types of inflammation [22–24]. Furthermore, baicalein and wogonin exerted obvious inhibitory effects on cancer as well as macrophages and angiogenesis [24, 25]. Paeoniflorin, one of major ingredients, could reduce lung metastasis of LLC through inhibiting the M2 activation [26]. In addition, the cytokines produced by activated innate immune cells in tumor microenvironment can stimulate tumorigenesis, such as IL-6 and TNF-*α* [27]. Our results showed a remarkable decrease in multiple proinflammatory cytokines such as TNF-a and IL-6 both in serum and tumor tissue of subcutaneous mouse model (Figure 2), suggesting the inhibitory effect of QRHX on cancer-related inflammation.

Tumor microenvironment, created by the tumor and mainly orchestrated by inflammatory cells, contributes to tumor escape, growth, progression, and evolution toward metastasis [28, 29]. Numerous studies in recent decade have presented evidences that TCM have a good effect on regulating tumor microenvironment, such as reversing the immunosuppressive microenvironment [11]. Macrophages, a basic component of the innate immune system, are infiltrated in virtually all malignancies. TAMs, M2-like polarized style, have been regarded as a protumor inflammatory microenvironment, which links inflammation and cancer [15]. Collective evidences demonstrate that TAMs have the ability of enhancing tumor angiogenesis, increasing migration and invasion, and suppressing the antitumor immune responses. It is correlated with the prognosis of patients with malignant tumor, such as lung cancer [30, 31]. Consistent with previous studies, in this study, we detected that the fraction of TAMs was increased in tumor and QRHX inhibited tumor growth in the tumor mice model through decreasing accumulating of TAMs and activation of M2.

CXCR4, which is widely expressed on malignant cells and binds to CXCL12 [32], plays an important role in hematopoiesis, development, and organization of the immune system by directly and indirectly mechanisms [33]. For example, in NSCLC, CXCL12-CXCR4 axis is involved in metastasis and associated with an unfavorable prognosis [34]; in ovarian cancer, it can control accumulation of human MDSCs and is an independent prognostic factor for tumor progression [35]. Several studies have reported that CXCL12 plays an important role in monocyte recruitment, differentiation, and function [18, 36]. In a mouse model of lung cancer, CXCL12 could recruit tumor-promoting myeloid CD11b+ cells [19]. Besides, powerful evidences indicate a role for CXCR4-CXCL12 axis in promoting macrophages polarization toward the M2 phenotype [37, 38]. In addition, M2 subpopulation is associated with angiogenic factors such as VEGF and CXCL12-CXCR4 axis can also promote tumor vascularization [39]. Excitedly, our results showed QRHX treatment inhibited signaling from tumor cells to macrophages through inducing a remarkable decrease in CXCL12 and CXCR4. Furthermore, QRHX inhibited angiogenesis likely through altering the tumor microenvironment by targeting TAMs.

In STAT family, there are seven proteins; STAT3 is one of them. It is a key transcription factor transducing signals from activated receptors or intracellular kinases to the nucleus and can be activated in tumor cells and immune cells [40]. In tumor, STAT3 could contribute to cancer development and progression, inhibit apoptosis of tumors, and help tumor escape immune system by suppressing the immune response [41]. Evidence indicated that when STAT3 binds to some receptor, it can be activated through Janus Kinases (JAKs), such as JAK2 [42]. Accumulating evidence implicates the important role of JAK2/STAT3 in tumor and macrophage polarization [27]. For instance, IL-6 can contribute to tumor cell survival and upregulate the antiapoptotic genes by driving JAK2/STAT3 signal [43]; IFN-*ɤ* activates macrophage by JAK-STAT signaling pathway [42]. What is more, JAK2 is associated with CXCR4 [39]. Then we can draw that TAMs in malignant tumors tend to M2 subtype possibly through CXCL12/CXCR4/JAK2/STAT3 signaling pathway. QRHX treatment blocked the response of macrophages to tumor signals by suppressing CXCL12/CXCR4/JAK2/STAT3 expression and induced a remarkable decrease of the recruitment of M2 macrophages, suggesting the attenuation of M2 subtype cells function by QRHX.

In TCM theory, TCM formulae have abundant medicinal materials and then regulate diseases through multitargets and multiways. Although the chemical constituents of QRHX have been separated and identified by HPLUNG CANCER-Q/TOF MS-UV method in our team (Supplementary Figure 1 and Table 1), the ingredients are various and their functions are enormous. So, there may be other mechanisms involved in macrophage polarization. What is more, there are diverse types cells in tumor, except for tumor cells and macrophage. Moreover, it is worth mentioning that TAMs were not separated and extracted from tumor tissue. CXCL12, CXCR4, JAK2, and STAT3 can not only play significant role in tumor cell or macrophage, but also other cells, such as T lymphocyte, neutrophils, tumor-associated fibroblast, and endothelial cells. Therefore, QRHX inhibited the CXCL12/CXCR4/JAK2/STAT3 axis possibly through other cells. Taken together, further investigations are needed to identify direct molecular targets of QRHX in macrophages in the context of cancer.

## 5. Conclusion

In conclusion, data from this study revealed that QRHX could suppress cancer progression by inhibiting the tumor promotion of TAMs in subcutaneous mice model, which could contribute to elucidating the underlying regulatory mode of QRHX on lung cancer treatment.

## Supplementary Material

Supplementary Figure 1, compounds in QRHX and Table 1, ion chromatography (A) of QRHX.

## Abbreviations

ELISA: Enzyme-linked immunosorbent assay
FITC: Fluorescein isothiocyanate
TNF-*α*: Tumor necrosis factor-*α*
IL-6: Interleukin-6
iNOS: Inducible nitric oxide synthase
VEGF: Vascular endothelial growth factor
QT-PCR: Quantitative Polymerase Chain Reaction
IF: Immunofluorescence
IHC: Immunohistochemical
TAMs: Tumor-associated macrophages
Arg-1: Arginase 1.

## References

[1] Ikonomidis I., Michalakeas C. A., Parissis J. (2012). Inflammatory markers in coronary artery disease. *BioFactors*.

[2] Lee P., Leung C. C., Restrepo M. I., Takahashi K., Song Y., Porcel J. M. (2016). Year in review 2015: lung cancer, pleural diseases, respiratory infections, bronchiectasis and tuberculosis, bronchoscopic intervention and imaging. *Respirology*.

[3] Sharma S. V., Bell D. W., Settleman J., Haber D. A. (2007). Epidermal growth factor receptor mutations in lung cancer. *Nature Reviews Cancer*.

[4] Leong D., Rai R., Nguyen B., Lee A., Yip D. (2014). Advances in adjuvant systemic therapy for non-small-cell lung cancer. *World Journal of Clinical Oncology*.

[5] Villanueva M. T. (2014). Microenvironment: the new midfielders in the tumour microenvironment. *Nature reviews. Cancer*.

[6] Mantovani A., Sozzani S., Locati M., Allavena P., Sica A. (2002). Macrophage polarization: tumor-associated macrophages as a paradigm for polarized M2 mononuclear phagocytes. *Trends in Immunology*.

[7] Mantovani A., Locati M. (2013). Tumor-associated macrophages as a paradigm of macrophage plasticity, diversity, and polarization lessons and open questions. *Arteriosclerosis, Thrombosis, and Vascular Biology*.

[8] Hu W., Li X., Zhang C., Yang Y., Jiang J., Wu C. (2016). Tumor-associated macrophages in cancers. *Clinical and Translational Oncology*.

[9] Qian B. Z., Pollard J. W. (2010). Macrophage diversity enhances tumor progression and metastasis. *Cell*.

[10] Mantovani A., Allavena P. (2015). The interaction of anticancer therapies with tumor-associated macrophages. *Journal of Experimental Medicine*.

[11] Xu J., Song Z., Guo Q., Li J. (2016). Synergistic effect and molecular mechanisms of traditional Chinese medicine on regulating tumor microenvironment and cancer cells. *BioMed Research International*.

[12] Wu J., Xu J., Eksioglu E. A. (2013). Icariside II induces apoptosis of melanoma cells through the downregulation of survival pathways. *Nutrition and Cancer*.

[13] Kong L., Liu J., Wang J. (2015). Icariin inhibits TNF-*α*/IFN-*γ* induced inflammatory response via inhibition of the substance P and p38-MAPK signaling pathway in human keratinocytes. *International Immunopharmacology*.

[14] Wu J., Du J., Xu C. (2010). In vivo and in vitro anti-inflammatory effects of a novel derivative of icariin. *Immunopharmacology and Immunotoxicology*.

[15] Conway E. M., Pikor L. A., Kung S. H. Y. (2016). Macrophages, inflammation, and lung cancer. *American Journal of Respiratory and Critical Care Medicine*.

[16] Shalapour S., Karin M. (2015). Immunity, inflammation, and cancer: an eternal fight between good and evil. *The Journal of Clinical Investigation*.

[17] Bingle L., Brown N. J., Lewis C. E. (2002). The role of tumour-associated macrophages in tumour progression: Implications for new anticancer therapies. *Journal of Pathology*.

[18] Kim D., Kim J., Yoon J. H. (2014). CXCL12 secreted from adipose tissue recruits macrophages and induces insulin resistance in mice. *Diabetologia*.

[19] Schmid M. C., Avraamides C. J., Foubert P. (2011). Combined blockade of integrin-*α*4*β*1 plus cytokines SDF-1*α* or IL-1*β* potently inhibits tumor inflammation and growth. *Cancer Research*.

[20] Balkwill F., Mantovani A. (2001). Inflammation and cancer: back to Virchow?. *The Lancet*.

[21] Aggarwal B. B., Shishodia S., Sandur S. K., Pandey M. K., Sethi G. (2006). Inflammation and cancer: how hot is the link?. *Biochemical Pharmacology*.

[22] Jiang W.-L., Chen X.-G., Zhu H.-B., Gao Y.-B., Tian J.-W., Fu F.-H. (2009). Paeoniflorin inhibits systemic inflammation and improves survival in experimental sepsis. *Basic and Clinical Pharmacology and Toxicology*.

[23] Kim I. D., Ha B. J. (2010). The effects of paeoniflorin on LPS-induced liver inflammatory reactions. *Archives of Pharmacal Research*.

[24] Fan G.-W., Zhang Y., Jiang X. (2013). Anti-inflammatory activity of baicalein in LPS-stimulated RAW264.7 macrophages via estrogen receptor and NF-*κ*B-dependent pathways. *Inflammation*.

[25] Li-Weber M. (2009). New therapeutic aspects of flavones: the anticancer properties of Scutellaria and its main active constituents Wogonin, Baicalein and Baicalin. *Cancer Treatment Reviews*.

[26] Wu Q., Chen G.-L., Li Y.-J., Chen Y., Lin F.-Z. (2015). Paeoniflorin inhibits macrophage-mediated lung cancer metastasis. *Chinese Journal of Natural Medicines*.

[27] Lin W., Karin M. (2007). A cytokine-mediated link between innate immunity, inflammation, and cancer. *Journal of Clinical Investigation*.

[28] Whiteside T. L. (2008). The tumor microenvironment and its role in promoting tumor growth. *Oncogene*.

[29] Lorusso G., Rüegg C. (2008). The tumor microenvironment and its contribution to tumor evolution toward metastasis. *Histochemistry and Cell Biology*.

[30] Ding L., Liang G., Yao Z. (2015). Metformin prevents cancer metastasis by inhibiting M2-like polarization of tumor associated macrophages. *Oncotarget*.

[31] Yuan A., Hsiao Y. J., Chen H. Y. (2015). Opposite effects of M1 and M2 macrophage subtypes on lung cancer progression. *Scientific Reports*.

[32] Balkwill F. (2004). Cancer and the chemokine network. *Nature Reviews Cancer*.

[33] Burger J. A., Kipps T. J. (2006). CXCR4: a key receptor in the crosstalk between tumor cells and their microenvironment. *Blood*.

[34] Phillips R. J., Burdick M. D., Lutz M., Belperio J. A., Keane M. P., Strieter R. M. (2003). The stromal derived factor-1/CXCL12-CXC chemokine receptor 4 biological axis in non-small cell lung cancer metastases. *American Journal of Respiratory and Critical Care Medicine*.

[35] Obermajer N., Muthuswamy R., Odunsi K., Edwards R. P., Kalinski P. (2011). PGE-induced CXCL 12 production and CXCR4 expression controls the accumulation of human MDSCs in ovarian cancer environment. *Cancer Research*.

[36] Sánchez-Martín L., Estecha A., Samaniego R., Sánchez-Ramón S., Vega M. Á., Sánchez-Mateos P. (2011). The chemokine CXCL12 regulates monocyte-macrophage differentiation and RUNX3 expression. *Blood*.

[37] Beider K., Bitner H., Leiba M. (2014). Multiple myeloma cells recruit tumor-supportive macrophages through the CXCR4/CXCL12 axis and promote their polarization toward the M2 phenotype. *Oncotarget*.

[38] Mota J. M., Leite C. A., Souza L. E. (2016). Post-sepsis state induces tumor-associated macrophage accumulation through CXCR4/CXCL12 and favors tumor progression in mice. *Cancer Immunology Research*.

[39] Teicher B. A., Fricker S. P. (2010). CXCL12 (SDF-1)/CXCR4 pathway in cancer. *Clinical Cancer Research*.

[40] Heinrich P. C., Behrmann I., Müller-Newen G., Schaper F., Graeve L. (1998). Interleukin-6-type cytokine signalling through the gp130/Jak/STAT pathway. *Biochemical Journal*.

[41] Kortylewski M., Kujawski M., Wang T. (2005). Inhibiting Stat3 signaling in the hematopoietic system elicits multicomponent antitumor immunity. *Nature Medicine*.

[42] Hu X., Chen J., Wang L., Ivashkiv L. B. (2007). Crosstalk among Jak-STAT, Toll-like receptor, and ITAM-dependent pathways in macrophage activation. *Journal of Leukocyte Biology*.

[43] Catlett-Falcone R., Landowski T. H., Oshiro M. M. (1999). Constitutive activation of Stat3 signaling confers resistance to apoptosis in human U266 myeloma cells. *Immunity*.

