# Supplementary material for: Targeting Tumor Microenvironment: Effects of Chinese Herbal Formulae on Macrophage-Mediated Lung Cancer in Mice
Source: Evid Based Complement Alternat Med. 2017 May 28;2017:7187168. doi: 10.1155/2017/7187168 (PMC5467330; doi:10.1155/2017/7187168)
Supplement: Supplementary file 1 — Supplementary Figure 1, compounds in QRHX and Table 1, ion chromatography (A) of QRHX. [file 7187168.f1.pdf]

Supplementary data

Table 1 The contents of the 8 bioactive compounds in Qingre Huoxue formula

Compound Name	No.	Concentration (mg/g)
Albiflorin	1	1.56± 0.00
Paeoniflorin	2	11.8 ± 0.0
Baicalin	3	36.2 ± 0.2
Oroxylin A-7-O-glucuronide	4	2.49± 0.03
Wogonoside	5	12.0 ± 0.1
Baicalein	6	6.36± 0.05
Wogomn	7	1.86± 0.02
Oroxylin A	8	0.606± 0.007

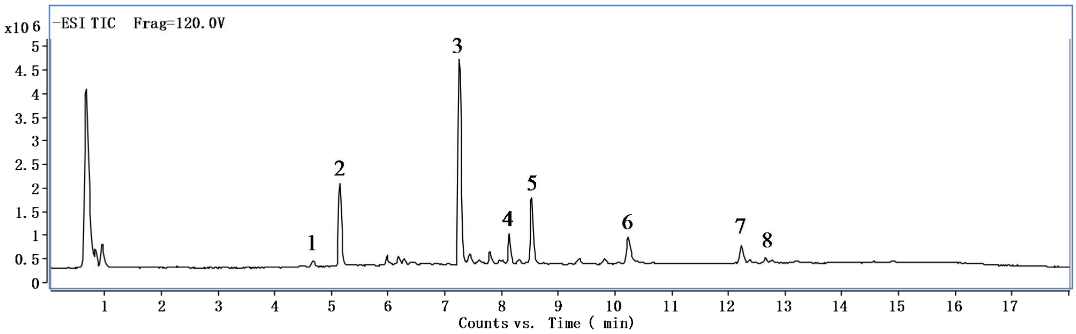

Figure 1.Total ion chromatography (A) of Qingre Huoxue formula by HPLC-Q/TOF MS
